# Psychosocial interventions for cardiac surgery patients: continuity at clinical stages

**DOI:** 10.1192/j.eurpsy.2022.1943

**Published:** 2022-09-01

**Authors:** O. Nikolaeva, T. Karavaeva, E. Nikolaev, N. Maksimova, E. Litvinova, E. Vasilieva

**Affiliations:** 1 Ulianov Chuvash State University, Department Of Faculty And Hospital Therapy, Cheboksary, Russian Federation; 2 Republican Cardiology Clinic, Cardiosurgery Unit, Cheboksary, Russian Federation; 3 St. Petersburg State University, Department Of Medical Psychology And Psychophysiology, St. Petersburg, Russian Federation; 4 V. M. Bekhterev National Medical Research Center for Psychiatry and Neurology, Department For Treatment Of Borderline Mental Disorders And Psychotherapy, St. Petersburg, Russian Federation; 5 St. Petersburg State Pediatric Medical University, Department Of General And Applied Psychology With A Course In Biomedical Disciplines, St. Petersburg, Russian Federation; 6 N.N. Petrov National Medical Research Center of Oncology, Scientific Department Of Innovative Methods Of Therapeutic Oncology And Rehabilitation, St. Petersburg, Russian Federation; 7 Ulianov Chuvash State University, Social And Clinical Psychology Department, Cheboksary, Russian Federation

**Keywords:** psychosocial interventions, perioperative period, psychological methods and techniques, cardiac surgery patients

## Abstract

**Introduction:**

More often, cardiac surgery patients (CSP) receive systematic psychological aid after surgery. However, their need for psychosocial interventions in the perioperative period is underestimated.

**Objectives:**

The goal is to determine the stages of psychosocial interventions for CSP that could cover the whole period of their treatment and rehabilitation.

**Methods:**

Analysis of scientific papers and practical experience gained in cardiologic clinic allowed dividing the system of psychosocial interventions for cardiac surgery patients into periods in accordance with actual stages of medical aid for CSPs.

**Results:**

According to the principles of personalized approach, we determined six consecutive semantically different stages of psychosocial interventions: out-of-hospital pre-surgery, in-hospital pre-surgery, early post-surgery, in-hospital post-surgery, post-surgery rehabilitation, and out-of-hospital rehabilitation. They have different duration and cover the whole period of treatment and rehabilitation of CSPs beginning with the moment of indication to surgery up to the complete rehabilitation and full adaptation to their post-surgery somatic condition. Each stage has its own goals, main objectives and expectations. Duration of the stages is conditional and can change depending on the nature of every clinical situation.

**Conclusions:**

Determination of clinical stages in the process of psychosocial interventions for CSPs gives ground for selecting optimal psychological methods and techniques for each stage and sets exact goals, achievement of which becomes possible only through a properly organised work of an interdisciplinary team of specialists.

**Disclosure:**

No significant relationships.

